# Interactions between caveolin 1 polymorphism and the Mediterranean and Mediterranean-DASH Intervention for Neurodegenerative Delay diet (MIND) diet on metabolic dyslipidemia in overweight and obese adult women: a cross-sectional study

**DOI:** 10.1186/s13104-021-05777-4

**Published:** 2021-09-20

**Authors:** Nasim Khatibi, Atieh Mirzababaei, Farideh Shiraseb, Faezeh Abaj, Fariba Koohdani, Khadijeh Mirzaei

**Affiliations:** 1 Shahid Sadoughi University of Medical Science, Yazd, Iran; 2 Department of Community Nutrition, School of Nutritional Sciences and Dietetics, Tehran University of Medical Sciences (TUMS), P.O. Box: 14155-6117, Tehran, Iran; 3 Department of Cellular, School of Nutritional Sciences and Dietetics, Tehran University of Medical Sciences (TUMS), Molecular Nutrition, Tehran, Iran

**Keywords:** MIND diet, Metabolic dyslipidemia, Caveolin 1, Obesity, Personalized nutrition

## Abstract

**Objective:**

The increased prevalence of metabolic dyslipidemia (MD) and its association with a variety of disorders raised a lot of attention to its management. Caveolin 1 (CAV1) the key protein in the caval structure of plasma membranes is many cell types that play an important role in its function. (CAV1) is a known gene associated with obesity. Today, a novel diet recognized as the Mediterranean and Mediterranean-DASH Intervention for Neurodegenerative Delay diet (MIND) is reported to have a positive effect on overall health. Hence, we aimed to investigate the interactions between CAV1 polymorphism and MIND diet on the MD in overweight and obese patients.

**Results:**

Remarkably, there was a significant interaction between the MIND diet and CAV1 rs3807992 for dyslipidemia (β = − 0.25 ± 132, P = 0.05) in the crude model. Whereby, subjects with dominant alleles had a lower risk of dyslipidemia and risk allele carriers with higher adherence to the MIND diet may exhibit the lower dyslipidemia. This study presented the CAV1 gene as a possible genetic marker in recognizing people at higher risks for metabolic diseases. It also indicated that using the MIND diet may help in improving dyslipidemia through providing a probable interaction with CAV1 rs3807992 polymorphism.

**Supplementary Information:**

The online version contains supplementary material available at 10.1186/s13104-021-05777-4.

## Introduction

Dyslipidemia is a metabolic disorder that imposes an enormous burden on public health [1]. Some concerns exist regarding “Metabolic dyslipidemia” (MD), with high triglyceride (TG) and low levels of high-density lipoprotein (HDL) cholesterol, which is associated with an elevated risk of Coronary heart disease (CHD) [2, 3]. Dyslipidemia is a significant primary risk factor for atherosclerosis, which is considerably prevalent in Iran. Moreover, people with central obesity and diabetes have a greater susceptibility to dyslipidemia [4].

As a multifactorial condition, MD and obesity are also determined by environmental conditions such as dietary intake and genetic variations [5]. In this manner, genetically related investigations have examined cases linked to complex diseases related to dyslipidemia from various populations [6, 7]. CAV1 has the ability to regulate various signals as well as maintain cholesterol homeostasis [8]. It is associated with cholesterol release and dyslipidemia risk factors [9].

The content of dietary patterns is important as controlling factors linked to the risk of dyslipidemia [10]. Some studies have investigated the interplays between Dietary Approaches to Stop Hypertension (DASH) diet and Mediterranean diet with dyslipidemia separately. It should be noted that high intakes of saturated fatty acids and unhealthful styles positively influence MD and the DASH diet is reported to have inverse associations with blood lipid concentrations [11]. A review on the correlation between dietary patterns and dyslipidemia also found that the DASH diet containing reduces the risk of MD [12]. The Mediterranean and Mediterranean-DASH intervention for MIND diet ingredients are replete with antioxidants that improve heart health and mitigate Hypertension (HTN) risk [13]. Another study examined the main role of the CAV1 gene in developing the cardiovascular disease with an effect on lipid factors and reported significant associations [14]. In this regard, we recently explored information about the interaction between CAV1 and dietary intake in overweight and obese women [15, 16].

To the authors’ knowledge, there has been no previous study evaluating the interaction between the MIND diet and CAV1 polymorphism towards dyslipidemia risk factors. Therefore, the present study intended to investigate the interaction between MIND diet with the CAV1 gene in association with MD.

## Main text

### Study population

In the present cross-sectional research, a random selection of referral patients was performed from health centers in Tehran, Iran. This study was approved by the Ethics Commission of Tehran University of Medical Sciences (IR.TUMS.VCR.REC.1398.142). Subjects included healthful overweight and non-menopause women aged over 18 years on average, with a BMI ranging from 25 to 40 kg/m^2^.

Women with a history of inflammatory disease, cardiovascular disease, diabetes mellitus, hypertension, kidney failure, stroke, thyroid disease, liver disease, cancer, and those who were on weight loss programs or reported daily energy intakes between 800 and 4200 kcal/day, or using supplements and medications during the study time were all excluded.

#### Biochemical assessment

All blood samples were collected after having an 8–12 h fasting state at the Nutrition and Genomics Laboratory of TUMS. Moreover, fasting blood sugar (FBS), TG, total cholesterol level, low-density lipoprotein (LDL), and HDL were measured according to standard protocols [17]. All of which were measured at the Bionanotechnology laboratory, Tehran University of Medical Science.

#### Anthropometric measurement and body composition

The anthropometric indices were measured for all participants. Weight (kg), Height (m), waist circumference (WC, cm), and the waist-to-hip ratio (WHR) were measured. Body mass index (BMI) was calculated as weight (kg) divided by height (m^2^). Respectively the researchers assessed the body composition of all cases with the use of Body Composition Analyzer BC-418MA-In Body (United Kingdom). The device calculates body fat percentage, fat mass, and fat-free mass (FFM) and predicts skeletal muscle mass (SMM) based on data obtained by dual-energy X-ray absorptiometry (DXA) using bioelectrical impedance analysis.

#### Dietary assessment

The diet scores were estimated using a semi-quantitative Food Frequency Questionnaires (FFQ) including a list of 147 food items this questionnaire has well-established reliability and validity specifically for Iranian adults [18, 19]. The software program, Nutritionist IV, was used for nutrient analysis and was modified for Iranian foods. A MIND diet score using the methodology described by Morris et al., focusing on 15 components is. There was a maximum of 15 points, higher intake of brain-healthy food groups, was scored 0, 0.5, or 1 point depending on the level of consumption [20].

#### Assessment of other variables

Assessment of physical activity was based on the International Physical Activity Questionnaire (IPAQ).

Low HDL ≤ 50 mg/dl and TG > 150 mg/dl indices were considered for metabolic dyslipidemia in participants [21].

#### DNA genotyping

For DNA extraction from whole blood by the Gene All Mini Columns Type kit, 1 ml of RBC lysis solution was initially decanted into a 2 ml microtube that contained 300 μl of the blood and subjected to gentle shaking 5 times, followed by overtaxing for 10 s and then centrifugation at 13,000 rpm for 3 min. Amplification of gene region containing CAV1 polymorphism (rs3807992) with G as the major allele (dominant allele) and A as the minor allele was conducted via the polymerase chain reaction-restriction fragment length polymorphism (PCR-RFLP) technique using the following primers:

- F: 3′AGTATTGACCTGATTTGCCATG5′, R: 5′GTCTTCTGGAAAAAGCACATGA-3′

The process of PCR reactions was conducted with an initial denaturation step at 94 °C for 3 min followed by 40 cycles of amplification including denaturation at 94 °C for 1 min, annealing at 42–50 °C for 1 min and elongation at 72 °C for 2 min.

#### Statistical analysis

Normality distribution was surveyed by applying Kolmogorov–Smirnov’s test. Data were analyzed by IBM SPSS version 22 (SPSS, Chicago, IL, USA). Quantitative variables were reported by (Mean ± SD) and qualitative variables were expressed using percentages and numbers. Comparison of quantitative and qualitative variables between genotypes and MIND diet was performed using one-way analysis of variance (ANOVA) and Chi-square test respectively. The analysis of covariance (ANCOVA) was performed after adjustment for age, total energy intake, BMI, and physical activity. Binary logistic regression was used for calculating the odds ratio and 95% confidence intervals (95% CI) for assessing the interaction between the MIND diet and genotypes on metabolic dyslipidemia.

### Results

#### Study population characteristics

This cross-sectional study was performed on 263 overweight and obese women within the range of 18–55 years old. The means (± SD) of age and BMI of individuals were 36.67 ± 9.10 and 161.2 ± 5.87 (kg/m^2^).

The frequencies of the G allele were 38%. The overall prevalence of CAV1 rs3807992 genotypes was 25.5%, 22.3% and 47.8% for AA, AG, and GG respectively Table 1.

**Table 1:** Cav1 rs3807992 genotypes and allelic variants of the study population

Cav1 rs3807992 genotypes	Genotypes frequency			Alleles frequency	
	GG	AG	AA	A	G
	47.8% (n = 193)	22.3% (n = 99)	25.5% (n = 103)	38.6%	61.4%

The demographic, anthropomorphic, and biochemical characteristics of participants across quartiles of MIND are shown in Table 2. After adjustment for BMI, age, total energy intake, and physical activity, there was a significant difference in FFM (P = 0.03). The individuals in the fourth quartile had higher FFM rather than the first quartile. Moreover, there is a significant association between dyslipidemia (P = 0.01) and TG (P = 0.00) across the quartiles. It is shown that individuals with dominant alleles had higher dyslipidemia and a higher level of TG. Also, there was a marginally significant difference between groups for IPAQ and SMM (P = 0.06).

**Table 2:** Participant characteristics consist of anthropometric measurements, and body composition, blood parameters across the quartiles of the MIND diet

Variables	Q1(n = 97)	Q2 (n = 98)	Q3 (n = 98)	Q4 (n = 98)	P-value	P-value*
	Mean ± SD					
Age (year)	35.52 ± 8.69	37.49 ± 9.72	36.88 ± 9.71	36.88 ± 8.67	0.49	0.48
Weight (kg)	80.82 ± 12.21	80.43 ± 13.49	81.01 ± 12.01	82.40 ± 11.31	0.69	0.27
Height (cm)	161.04 ± 5.46	161.26 ± 5.96	161.20 ± 6.00	161.08 ± 6.17	0.99	0.63
IPAC (MET-minutes/week)	1007.75 ± 1754.61	785.62 ± 588.73	1339.00 ± 2699.21	1541.56 ± 2468.50	0.16	0.06
Body composition						
BMI (kg/m^2^)	31.29 ± 4.49	30.87 ± 4.64	31.22 ± 4.28	31.68 ± 3.77	0.62	0.35
SMM (kg)	25.19 ± 2.99	25.44 ± 3.54	25.51 ± 3.60	26.02 ± 3.49	0.38	0.06
FFM (kg)	45.99 ± 5.01	46.19 ± 5.74	46.57 ± 6.03	47.23 ± 5.83	0.44	**0.03**
BFM (%)	35.13 ± 9.15	34.15 ± 9.82	34.54 ± 8.55	35.09 ± 7.37	0.83	0.87
WHR (%)	1.87 ± 9.24	0.93 ± 0.04	0.94 ± 0.05	0.93 ± 0.05	0.39	0.55
WC (cm)	99.43 ± 9.87	98.63 ± 10.54	99.80 ± 10.55	100.48 ± 9.33	0.63	0.27
PBF (%)	42.66 ± 5.65	42.04 ± 5.54	42.16 ± 5.51	42.03 ± 5.36	0.83	0.30
Blood pressure						
SBP (mmHg)	108.97 ± 17.22	112.77 ± 13.26	112.62 ± 14.61	111.07 ± 14.39	0.41	0.96
DBP (mmHg)	76.37 ± 12.63	79.75 ± 9.37	77.75 ± 9.74	76.39 ± 9.62	0.17	0.35
Biochemical assessment						
FBS (mg/dl)	86.30 ± 9.75	87.23 ± 9.08	88.20 ± 11.09	87.97 ± 8.76	0.72	0.77
TG (mg/dl)	124.33 ± 57.90	118.65 ± 65.13	121.53 ± 58.80	93.19 ± 50.9	0.13	0.45
HDL (mg/dl)	45.03 ± 9.16	48.43 ± 10.65	45.45 ± 9.77	47.83 ± 12.66	0.22	0.38
LDL (mg/dl)	90.67 ± 22.52	97.45 ± 24.92	94.53 ± 24.12	96.54 ± 24.82	0.46	0.49
HOMA-IR	3.33 ± 1.30	3.44 ± 1.35	3.38 ± 1.28	2.91 ± 0.89	0.34	0.92
Insulin (mIU/ml)	1.24 ± 0.22	1.18 ± 0.24	1.20 ± 0.24	1.22 ± 0.20	0.50	0.76
hs.CRP (mg/l)	4.59 ± 5.10	4.16 ± 4.51	3.92 ± 4.06	4.56 ± 4.93	0.84	0.61
ALT (mg/dl)	17.67 ± 7.10	17.30 ± 7.52	17.51 ± 7.47	18.60 ± 7.39	0.73	0.36
AST (mg/dl)	19.63 ± 13.36	17.43 ± 11.80	19.65 ± 14.16	19.81 ± 12.76	0.70	0.81
Cholesterol (mg/dl)	178.55 ± 38.37	190.16 ± 33.45	181.55 ± 37.48	188.63 ± 35.59	0.24	0.57
MIND-score quartile						
AA%	28.7%	23.8%	28.7%	18.8%	0.67	0.45
AG%	15.9%	28.0%	25.6%	30.5%		
GG%	26.8%	24.7%	22.6%	25.8%		
Dyslipidemia						
Without	85 (87.6%)	73 (74.5%)	71 (72.4%)	60 (61.2%)	**0.00**	0.01
With	12 (12.4%)	25 (25.5%)	27 (27.6%)	38 (38.8%)		
HDL (mg/dl)						
< 50	63 (64.9%)	61 (62.2%)	58 (59.2%)	51 (52.0%)	0.06	0.74
≥ 50	34 (21.5%)	37 (23.4%)	40 (25.3%)	47 (29.7%)		
TG (mg/dl)						
< 150	66 (68.0%)	50 (51.0%)	47 (48.0%)	24 (24.5%)	**0.00**	**0.00**
≥ 150	31 (32.0%	48 (49.0%)	51 (52.0%)	74 (75.5%)		
Marital status						
Single	28 (25.9%)	26 (24.1%)	28 (25.9%)	26 (25.9%)	0.59	0.90
Married	68 (24.2%)	71 (25.3%)	70 (24.9%)	72 (25.6%)		
Educational level						
Illiterate	0 (0.0%)	0 (0.0%)	2 (50.0%)	2 (50.0%)	0.61	0.27
Underdiploma	9 (18.4%)	14 (28.6%)	14 (28.6%)	12 (24.5%)		
College education	87 (25.9%)	83 (24.7%)	82 (24.4%)	84 (25.0%)		
Economic status						
Low	9 (22.5%)	9 (22.5%)	10 (25.0%)	12 (30.0%)	0.47	0.91
Moderate	42 (25.3%)	51 (30.7%)	36 (21.7%)	37 (22.3%)		
Good	40 (26.3%)	31 (20.4%)	41 (27.0%)	40 (26.3%)		
Excellent	4 (21.1%)	4 (21.1%)	7 (36.8%)	4 (21.1%)		

Bold values of table are significantly different from zero at *P* < 0.005

Quantitative variables were reported with mean and SD and qualitative variables with number and percentage values were calculated by ANOVA as mean ± SD

Variables are presented by mean ± SD for continuous variables and frequency for categorical variables

MD: metabolic dyslipidemia: TG > 150 and HDL < 40

*BMI* body mass index, *WC* waist circumference, *WHR*, waist-to-hip ratio, *FFM* fat-free mass, *HDL* high-density lipoprotein, *hs-CRP* high-sensitivity C reactive protein, *LDL* low-density lipoprotein, *BMR* basal metabolic rate, *TG* triacylglycerol, *TC* total cholesterol, *SBP* systolic blood pressure, *DBP* diastolic blood pressure, *ALT* alanine transaminase, *AST* aspartate transaminase, *IPAC* international physical activity questionnaire, *PBF* percent body fat, *BFM* body fat mass, *SMM* skeletal muscle mass

P values resulted from the analysis of one-way ANOVA for continuous variables and chi-square test for categorical variables. Tukey test was performed to compare each genotype with other types for continuous variables

*P-value is found by ANCOVA and adjusted for age, BMI, physical activity, and total energy intake

#### Investigation of body composition, biochemical variables, and RMRs among the CAV1 rs3807992 genotypes

Additional file 1: Table S1 shows the association between anthropometric body composition, biochemical parameters, and CAV1 rs3807992 genotypes. We observed that the individuals who had GG alleles had a higher level of DBP (P = 0.02). There is also a meaningful association between genotype and two categories of TG (P = 0.01). It was seen that 52.3% of GG carriers had TG < 150. It was seen that individuals with the higher carrier of AA had higher weight and lower levels of DBP rather than other groups.

#### Investigation of dietary intake among the CAV1 rs3807992 genotypes

The food group and nutrient intakes according to CAV1 rs3807992 genotypes are shown in (Additional file 1: Table S2). Significant differences were seen in other vegetable and fast food groups (P < 0.05).

#### Interaction of MIND diet and CAV1 rs3807992 with dyslipidemia

Interaction between the MIND diet and CAV1 rs3807992 gene variants on dyslipidemia is shown in (Additional file 1: Table S3). There was a significant interaction between MIND diet and genotype for metabolic dyslipidemia (β = − 0.25 ± 132, OR = 0.77, 95% CI = 0.60–1.00, P = 0.05) in the crude model. Whereby, subjects with dominant allele had a lower risk of dyslipidemia. Besides, in model one age, IPAC, BMI, and energy intake had been controlled for participants had (β = − 0.34 ± 152, OR = 0.70, 95% CI = 0.52–0.95, P = 0.02), after controlling for age, IPAC, BMI, energy intake and job was observed subjects to have 0.64-fold in model two (β = − 0.44 ± 165, OR = 0.64, 95% CI = 0.46–0.88, P = 0.007), this inverse association becomes more significant.

**Fig. 1:**
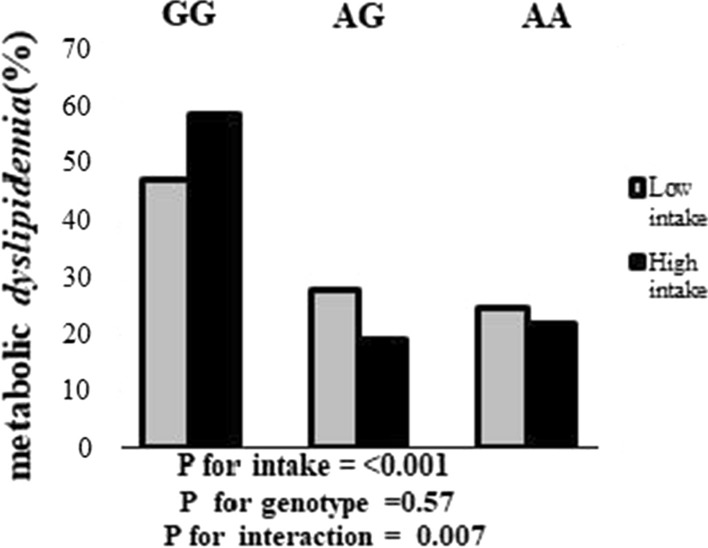
Percentage of Metabolic dyslipidemia across GG, AG, and AA genotypes based on intake of the MIND diet

Percentage of Metabolic dyslipidemia across CAV1 rs3807992 genotypes based on a low and high intake of the MIND diet. The percentage of Metabolic dyslipidemia in low intake across GG, AG, and AA genotypes were respiratory—%, 27.8%, and 25%. The percentage of Metabolic dyslipidemia in high intake across GG, AG, and AA genotypes were respiratory—%, 19%, and 22.2% (Fig. 1).

### Discussion

In this study, we reported the novel finding that AA carriers of rs3807992 CAV1 gene variant had higher weight rather than other groups. In this manner, we should note that CAV1 is a main fatty acid-binding protein in adipocytes [22, 23]. CAV1 also directly binds to cholesterol. A high amount of cholesterol is one of the hallmarks of the biogenesis of specialized membrane lipid rafts, called caveolae [24].

Recent studies exhibited that CAV1 can be transmitted to lipid droplets. It seems that the activity of CAV1is essential to preserve the perilipin function and the following lipid droplet integrity, thus its absence can result in the alterations of lipid droplet size [25, 26].

Previously, variants in the CAV1 gene have been connected to lipodystrophy, a disorder of unusual lipid distribution [27]. Today, various genome-wide studies have supported the correlation between the CAV1 variants and dyslipidemia, for instance, the low HDL and high TGs [28, 29]. Previous studies also showed the role of CAV1 in cardiometabolic disorders*.* These results were also reported in human studies with CAV1 mutations that exhibit dyslipidemia, insulin resistance, and diabetes [30–33]. It is demonstrated that another variant of the CAV1 gene means rs926198 is linked to dyslipidemia, particularly low HDL cholesterol and also other metabolic disorders, including diabetes, insulin resistance, metabolic syndrome, and cardiovascular risk in Caucasians and Hispanics. Therefore, it may be considered as a marker for cardiometabolic risk factors in non-obese people [34].

Remarkably, both knockout and autosomal recessive mutations in the CAV1 gene correlate with alterations in lipid and glucose metabolism despite a lean phenotype with reduced adiposity [31, 32]. Here we also elucidated that there was no significant association between all of the nutrients intakes across the three alleles of CAV1rs3807992 genotypes. There was a significant interaction between MIND diet and genotype for dyslipidemia and the AA carriers with higher adherence to the MIND diet may exhibit lower dyslipidemia. Nevertheless, there was no remarkable interaction between MIND diet and genotype on dyslipidemia after adjustments.

We also find a significant association between CAV1 genotypes with DBP, which remained significant after adjustment for age, BMI, physical activity, and total energy intake. Interestingly, AA carrier was associated with higher weight and lower DBP. However, another study on a Caucasian cohort with subsequent replication in a Hispanic cohort did not observe any relationship between the CAV1 variant and HTN [34].

### Conclusion

The CAV1 gene seems to be a genetic marker that might help in recognizing people at higher risks for metabolic diseases. The present study indicates that using a novel diet as a MIND diet may help in improving dyslipidemia by providing a possible interaction with CAV1 rs3807992 gene variants. Finally, more large-scale clinical studies with longitudinal data are necessary to confirm our data and to investigate other available diets in this field of research.

## Limitation

A food-frequency questionnaire (FFQ) was used to assess dietary intake that is self-reported and accordingly, reliant on the patient’s memory. This research focused primarily on the composition of MIND diet. However, other dietary patterns can also contribute to the progression of MD. Finally, because this is an observational study, the relationships revealed in Iranian women may not be applicable for people of other races.

## Supplementary Information

**Additional file 1: Table S1.** Participant characteristics consist of anthropometric measurement, body composition, and blood parameters across Cav1 rs3807992genotypes. **Table S2.** Dietary intake of study population according to Cav1 rs3807992 genotypes. **Table S3.** The interactions between mind diet and Cav1 rs3807992 genotype on the risk of MD.

## Data Availability

The data are not publicly available due to containing private information of participants. Data are however available from the authors upon reasonable request and with permission of Khadijeh Mirzaei.

## Abbreviations

ANOVA: One-way analysis of variance
ANCOVA: Analysis of covariance
BMI: Body mass index
CAV1: Caveolin 1
CHD: Coronary heart disease
CHD: Coronary heart disease
DASH: Dietary Approaches to Stop Hypertension
DXA: Dual-energy X-ray absorptiometry
DBP: Diastolic blood pressure
FBS: Fasting blood sugar
FFQ: Food Frequency Questionnaires
FFM: Fat-free mass
HTN: Hypertension
HDL: High-density lipoprotein
IPAQ: International Physical Activity Questionnaire
LDL: Low-density lipoprotein
MIND: Mediterranean-DASH Intervention for Neurodegenerative Delay diet
MD: Metabolic dyslipidemia
LDL: Low-density lipoprotein
PCR-RFLP: Polymerase chain reaction-restriction fragment length polymorphism
SMM: Skeletal muscle mass
TG: Triglycerides
WC: Waist circumference
WHR: Waist-to-hip ratio
WC: Waist circumference
WHR: Waist-to-height ratio
