# Supplementary material for: Interactions between caveolin 1 polymorphism and the Mediterranean and Mediterranean-DASH Intervention for Neurodegenerative Delay diet (MIND) diet on metabolic dyslipidemia in overweight and obese adult women: a cross-sectional study
Source: BMC Res Notes. 2021 Sep 20;14:364. doi: 10.1186/s13104-021-05777-4 (PMC8454002; doi:10.1186/s13104-021-05777-4)
Supplement: Supplementary file 1 — Additional file 1: Table S1. Participant characteristics consist of anthropometric measurement, body composition, and blood parameters across Cav1 rs3807992genotypes. Table S2. Dietary intake of study population according to Cav1 rs3807992 genotypes. Table S3. The interactions between mind diet and Cav1 rs3807992 genotype on the risk of MD. [file 13104_2021_5777_MOESM1_ESM.docx]

**Table S1. Participant characteristics cosist of anthropometric measurement, body composition, and blood parameters across Cav1 rs3807992genotypes**

| **P value*** | **P-value** | **AA=(103)** | **AG=(90)** | **GG=(193)** | **Cav1 rs3807992**  **Genotype** |
| --- | --- | --- | --- | --- | --- |
|  |  | **Mean ± SD** | | |  |
| 0.99 | 0.69 | 36.15±8.93 | 37.26±8.96 | 36.64±9.04 | Age(year) |
| **0.09** | 0.75 | 82.04±13.73 | 80.67±13.17 | 81.33±11.45 | Weight(kg) |
| 0.18 | 0.10 | 160.16±5.53 | 161.36±5.66 | 161.6±5.97 | Height(cm) |
| 0.82 | 0.79 | 1100.69±2468.81 | 1088.63±1034.32 | 1281.67±2310.61 | IPAC(**MET-minutes/week**) |
| **Body composition** | | | | | |
| 0.73 | 0.17 | 31.97±4.87 | 30.91±4.18 | 31.12±4.01 | BMI(kg/m^2^) |
| 0.12 | 0.14 | 25.01±3.04 | 25.60±3.72 | 25.84±3.46 | SMM(kg) |
| 0.17 | 0.22 | 45.71±5.19 | 46.69±6.23 | 46.89±5.64 | FFM(kg) |
| 0.58 | 0.10 | 36.35±9.96 | 33.94±8.51 | 34.40±8.14 | BFM(kg) |
| 0.12 | 0.25 | 1.82±9.01 | 0.93±0.05 | 0.93±0.04 | WHR |
| 0.49 | 0.71 | 100.23±10.88 | 99.04±10.42 | 99.74±9.54 | WC(cm) |
| 0.14 | 0.006 | 43.73±5.46 | 41.66±4.92 | 41.76±5.51 | PBF (%) |
| **Blood pressure** | | | | | |
| 0.16 | 0.60 | 109.77±13.26 | 111.55±17.92 | 111.90±14.24 | SBP(mmHg) |
| **0.02** | 0.84 | 75.28±9.37 | 77.41±12.91 | 78.62±9.41 | DBP(mmHg) |
| **Biochemical assessment** | | | | | |
| 0.43 | 0.17 | 89.03±10.56 | 85.80±8.44 | 87.68±9.88 | FBS(mmol) |
| 0.23 | 0.23 | 117.77±64.17 | 131.32±61.74 | 114.52±54.64 | TG (mg/dL) |
| 0.57 | 0.14 | 46.14±9.96 | 49.00±12.76 | 45.73±10.33 | HDL(mg/dL) |
| 0.46 | 0.43 | 91.92±24.91 | 97.45±25.58 | 95.21±22.99 | LDL(mg/dL) |
| 0.68 | 0.73 | 1.21±0.25 | 3.39±1.37 | 3.30±1.23 | HOMA IR |
| 0.98 | 0.57 | 1.21±0.25 | 1.25±0.23 | 1.21±0.22 | Insulin (mIU/ ml) |
| 0.52 | 0.52 | 4.14±4.57 | 3.86±4.01 | 4.66±4.97 | hs.CRP(mg/L) |
| 0.85 | 0.78 | 18.70±10.19 | 17.95±7.90 | 17.87±6.31 | ALT( mg/L) |
| 0.56 | 0.16 | 22.42±18.60 | 18.27±14.53 | 18.76±10.29 | AST (mg/L) |
| 0.49 | 0.72 | 182.39±38.05 | 186.28±36.42 | 87.56±9.76 | Cholesterol(mg) |
| **Dyslipidemia** | | | | | |
| 0.75 | 0.29 | 80(28.0%) | 67(23.4%) | 139(48.6%) | Without |
|  |  | 23(23.0%) | 23(23.0%) | 54(54.0%) | With |
|  | | | | | |
| **TG(mg/dl)** | | | | | |
| 0.01 | 0.65 | 53(51.5%) | 36(40.0%) | 101(52.3%) | <150 |
|  |  | 50 (48.5%) | 54(60.0%) | 92(47.7%) | ≥150 |
| **HDL(mg/dl)** | | | | | |
| 0.37 | 0.24 | 63(61.2%) | 56(62.2%) | 106(54.9%) | <50 |
|  |  | 40(38.8%) | 34(37.8%) | 87(45.1%) | ≥50 |
| **Marital state** | | | | | |
| 0.41 | 0.06 | 33(32.4%) | 26(25.5%) | 43(42.2%) | **Single** |
|  |  | 69(25.1%) | 59(21.5%) | 147(53.5%) | **Married** |
| **Educational status** | | | | | |
| 0.27 | 0.50 | 4(100.0%) | 0(0.0%) | 0(0.0%) | **Illiterate** |
|  |  | 12(24.5%) | 8(16.3%) | 29(59.2%) | **≤Diploma** |
|  |  | 86(26.5%) | 77(23.8%) | 161(49.7%) | **College education** |
| **Economics status** | | | | | |
| 0.70 | 0.90 | 10(27.0%) | 7(18.9%) | 20(54.1%) | **Low** |
|  |  | 45(27.3%) | 37(22.4%) | 83(50.3%) | **Moderate** |
|  |  | 34(23.8%) | 34(23.8%) | 75(52.4%) | **Good** |
|  |  | 7(35.0%) | 4(20.0%) | 9(45.0%) | **Excellent** |

MD:metabolic dyslipidemia: TG>150 and HDL<40

BMI, body mass index; WC, waist circumference; WHR, waist-to-hip ratio; FFM, fat-free mass; HDL, high-density lipoprotein; hs-CRP, high-sensitivity C reactive protein; LDL, low-density lipoprotein; BMR, basal metabolic rate; TG, triacylglycerol; TC, total cholesterol; SBP, systolic blood pressure; DBP, diastolic blood pressure, ALT: alanine transaminase,AST: aspartate transaminase, IPAC:international physical activity questionnaire ,PBF: percent body fat, BFM:body fat mass, SMM: skeletal muscle mass.

Quantitative variables were reported with mean and SD and qualitative variables with number and percentage. values were calculated by ANOVA as Mean±SD

Variables is presented by mean+SD for continuous variables and frequency for categorical variables

P values resulted from the analysis of one-way ANOVA for continuous variables and chi-square test for categorical variables. Tukey test was performed to compare each genotype with other types for continuous variables.

*P-value is found by ANCOVA and adjusted for age, BMI, physical activity, and total energy intake

**Table S2. Dietary intake of study population according to Cav1 rs3807992 genotypes**

| **P value*** | **P value** | **AA=103**  **Mean ± SD** | **AG=99**  **Mean ± SD** | **GG=193**  **Mean ± SD** | **Cav1 rs3807992**  **genotype** |
| --- | --- | --- | --- | --- | --- |
| **Macronutrient** | | | | | |
| _ | 0.28 | 2718.48±937.07 | 2526.97±764.28 | 2635.79±753.85 | Energy(kcal /day) |
| 0.46 | 0.42 | 94.57±38.68 | 88.78±30.50 | 90.51±27.56 | Protein(g/d) |
| 0.94 | 0. 51 | 382.39±130.68 | 361.25±125.63 | 371.05±119.92 | Carbohydrate (g /d) |
| **Micronutrient** | | | | | |
| 0.54 | 0.19 | 98.45±43.05 | 89.32±28.12 | 96.46±33.74 | Trans.fat(g /d) |
| 0.40 | 0.64 | 264.89±122.70 | 254.07±103.44 | 268.26±114.59 | Cholesterol(mg/d) |
| 0.25 | 0.09 | 29.69±13.74 | 26.12±8.98 | 28.91±11.29 | Saturated Fatty acid(g /d) |
| 0.81 | 0.35 | 32.98±15.54 | 30.30±10.91 | 32.34±12.48 | MUFA(g /d) |
| 0.99 | 0.57 | 20.78±11.40 | 19.27±8.10 | 20.10±9.33 | PUFA(g/d) |
| 0.20 | 0.18 | 0.00±0.00 | 0.00±0.00 | 0.00±0.00 | Trans fatty acid(g /d) |
| 0.69 | 0.60 | 49.04±24.47 | 46.48±20.95 | 46.58±19.53 | Dietary fiber(g /d) |
| **Mineral** | | | | | |
| 0.21 | 0.90 | 479.53±190.53 | 471.46±160.99 | 470.73±160.19 | Mg(mg/d) |
| 0.78 | 0.62 | 13.67±5.97 | 12.97±4.66 | 13.39±4.26 | Zinc(mg/d) |
| 0.73 | 0.59 | 2.08±0. 88 | 1.97±0.68 | 2.00±0.70 | Copper(mg/d) |
| 0.99 | 0.53 | 1304.28±635.96 | 1216.30±563.07 | 1268.03±447.79 | Calcium(mg/d) |
| 0.55 | 0.37 | 28.55±25.15 | 25.28±21.24 | 25.20±17.00 | Iron(mg/d) |
| 0.17 | 0.24 | 4730.70±2077.62 | 4485.97±1983.29 | 4362.92±1455.35 | Sodium(mg/d) |
| **Vitamins** | | | | | |
| 0.14 | 0.26 | 1.77±1.40 | 1.91±1.46 | 2.08±1.69 | D(μg/d) |
| 0.47 | 0.76 | 16.62±52 | 16.80±9.15 | 17.39±9.57 | E(mg/d) |
| 0.63 | 0.30 | 2.22±0.81 | 2.06±0.75 | 2.12±0.66 | B1(mg/d) |
| 0.48 | 0.34 | 2.30±0.96 | 2.15±0.89 | 2.31±0.79 | B2(mg/d) |
| 0.18 | 0.09 | 28.12±12.34 | 25.15±8.95 | 25.90±9.23 | B3(mg/d) |
| 0.44 | 0.24 | 2.30±0.88 | 2.12±0.69 | 2.16±0.70 | B6(mg/d) |
| 0.65 | 0.29 | 642.52±212.98 | 615.50±191.38 | 605.66±179.48 | B9(μg/d) |
| 0.58 | 0.85 | 4.30±2.59 | 4.28±2.84 | 4.44±2.34 | B12(μg/d) |
| 0.62 | 0.59 | 185.55±103.22 | 175.63±97.25 | 191.34±131.11 | ‍C(mg/d) |
| 0.12 | 0.12 | 6.18±1.63 | 6.72±1.63 | 6.42±1.87 | Mind Score |
| **Component of Mind Score** | | | | | |
| 0.35 | 0.49 | 0.48±0.39 | 0.54±0.40 | 0.49±0.40 | Wholegrain(g/day) |
| 0.53 | 0.62 | 0.49±0.38 | 0.48±0.37 | 0.45±0.39 | Poultry(g/d) |
| 0.33 | 0.53 | 0.47±0.40 | 0.54±0.42 | 0.49±0.40 | Beans(g/d) |
| 0.70 | 0.88 | 0.48±0.40 | 0.50±0.41 | 0.51±0.40 | Nuts(g/d) |
| 0.15 | 0.22 | 0.43±0.40 | 0.53±0.37 | 0.47±0.38 | Fishes(g/d) |
| 0.12 | 0.06 | 0.51±0.41 | 0.40±0.41 | 0.52±0.39 | Berries(g/d) |
| 0.31 | 0.54 | 0.47±0.40 | 0.53±0.40 | 0.48±0.40 | Green Vegetable(g/d) |
| 0.03 | 0.07 | 0.53±0.40 | 0.56±0.40 | 0.45±0.41 | Other vegetable(g/d) |
| 0.63 | 0.52 | 0.44±0.43 | 0.50±0.41 | 0.49±0.41 | Butter(g/d) |
| 0.88 | 0.83 | 0.51±0.42 | 0.51±0.41 | 0.50±0.38 | Cheese(g/d) |
| 0.35 | 0.80 | 0.51±0.42 | 0.47±0.38 | 0.50±0.41 | Red meat(g/d) |
| 0.01 | **0.005** | 0.39±0.40 | 0.55±0.41 | 0.53±0.39 | Fast-food(g/d) |
| 0.52 | 0.26 | 0.44±0.40 | 0.53±0.38 | 0.51±0.42 | Sweets(g/d) |

MD:metabolic dyslipidemia: TG>150 and HDL<40

After adjustment for calorie intake. Variables are presented by mean+SD.P values resulted from the analysis of one-way ANOVA.P-value* is obtained by ANCOVA after adjustment for calories intake.

**Table S3. The interactions between mind diet and Cav1 rs3807992 genotype on the risk of MD**

| **P-value** | **OR(95% C.I)** | **β±SE** |  |
| --- | --- | --- | --- |
| 0.05 | 0.77(0.60-1.00) | -0.25±132 | Crude model |
| 0.02 | 0.70(0.52-0.95) | -0.34±152 | Model 1 |
| 0.007 | 0.64(0.46-0.88) | -0.44±165 | Model 2 |

MD:metabolic dyslipidemia: TG>150 and HDL<40

Crude Model: In this model, the effect of any of the confounders is not modified

Model 1: In this model, the effect of age, BMI, energy intake, and physical activity is adjusted

Model 2: In this model, for adjustment model 1 and job is adjusted

p-value≤0.05
